# Success of supportive periodontal therapy in periodontitis patients — A retrospective analysis

**DOI:** 10.1111/idh.12521

**Published:** 2021-08-04

**Authors:** Max G. P. Schoenmakers, Eveline J. S. Willems, Dagmar Else Slot, G. A. (Fridus) Van der Weijden

**Affiliations:** ^1^ Department of Periodontology Academic Centre for Dentistry Amsterdam (ACTA) A Joint Venture between the Faculty of Dentistry of the University of Amsterdam and the Faculty of Dentistry of the Vrije Universiteit Amsterdam Amsterdam The Netherlands; ^2^ Clinic for Periodontology Utrecht The Netherlands

**Keywords:** active periodontal therapy, bleeding on probing, periodontal treatment, probing pocket depth, supportive periodontal therapy, tooth loss

## Abstract

**Objective:**

The aim of this retrospective analysis was to evaluate, in adult patients treated for periodontitis, the periodontal stability during supportive periodontal therapy (SPT).

**Methods:**

Data were collected and analyzed retrospectively for periodontitis patients aged ≥36 years who underwent active periodontal therapy (APT) and were following an SPT programme. The stability of the APT success, defined as a probing pocket depth (PPD) of ≤5 mm, was the main outcome parameter. Analyses were performed in which PPD, tooth loss (TL), bleeding on probing (BOP), periodontal epithelium surface area (PESA), and the effects of age, gender, smoking status, and the number of years in SPT were evaluated. The annual TL and BOP of <10% in addition to a PPD of ≤5 mm were considered to be secondary outcome variables.

**Results:**

In total, 993 patients were included, in 36% of whom a PPD ≤5 mm was found at the evaluation of APT. If the outcome was defined as a BOP of <10% in addition to a PPD of ≤5 mm, this was present in only 16% of the patients. During SPT, a small overall increase in clinical parameters for the total population and an annual average TL of 0.15 per patient was observed. Patients of male gender and smokers negatively affected the success of SPT.

**Conclusion:**

The periodontal clinical status remained ‘fairly’ stable during SPT in chronic periodontitis patients aged ≥36 years. Smoking negatively affects the outcome of APT and periodontal stability during SPT.

## 1 INTRODUCTION

Periodontitis is a chronic inflammatory disease primarily characterized by signs such as swollen and red gingiva, gingival bleeding, periodontal pocketing, irreversible progressive loss of connective tissue attachment, and horizontal and/or angular alveolar bone loss due to inflammation.^1^, ^2^, ^3^ Periodontitis is a worldwide health concern. Kassebaum et al.^4^, ^5^ showed that the global prevalence of severe periodontitis is 7–11%. This makes it the sixth most common disease in 2010.^4^ The number of people affected by periodontitis increases with age and reaches a precipitous increase at the age of 38 years. It subsequently stabilizes and remains constant in older age groups.^4^ Of all tooth loss (TL), 30–35% is due to periodontitis. Periodontitis results in problems with chewing, aesthetics and systematic inflammation, which means that it has a great impact on the quality of life of those affected.^2^, ^3^, ^6^

The first step in treating periodontitis is active periodontal therapy (APT). This therapy is designed to preserve patients’ natural dentition and supports oral health.^3^ To ensure long‐term stability of the periodontium after APT, patients are entered into a supportive periodontal therapy (SPT) programme to reduce the possibility of reinfection and further development of periodontitis.^7^ An important focus area during SPT is supragingival plaque control, as this has proven to maintain the obtained stable periodontal condition.^8^, ^9^ As periodontitis is a multifactorial disease, the interaction between different factors contributing to individual susceptibility for developing the disease may influence patients’ response to periodontal therapy and their stability during SPT.^3^, ^10^ Even after the completion of periodontal therapy with the most optimal results, life‐long supportive care to prevent the recurrence of periodontitis is recommended.^7^ Numerous studies have shown that SPT is an effective way to maintain a healthy and stable periodontium in the long term and to prevent TL by controlling the dental plaque level.^11^, ^12^, ^13^ However, long‐term research into SPT using clinical periodontal parameters, such as bleeding on probing (BOP), probing pocket depth (PPD) and TL, is limited. Other researchers have stated that the use of parameters—such as periodontal epithelial surface area (PESA), which quantifies the surface area of the pocket epithelium if marginal gingiva is at or below the cementoenamel junction—could serve as surrogate markers for systemic endpoints.^14^, ^15^ These clinical parameters should be assessed in combination with supplementary variables that could affect the success of the SPT, such as age, gender, smoking status and the frequency and duration of SPT.^16^, ^17^, ^18^, ^19^

The ultimate goal of SPT is to support patients’ oral health and maintain the treatment results achieved at the evaluation of APT.^7^ The aim of this practice‐based retrospective analysis is to evaluate, based on endpoint parameters, the periodontal stability during SPT in treated periodontitis patients.

## 2 MATERIAL AND METHODS

### 2.1 Source of Data and Participants

This report is an observational retrospective analysis.^20^ The STROBE and RECORD checklists for observational studies were used.^21^, ^22^ During 2017, data available from digital treatment records were procured retrospectively by staff members of the Clinic for Periodontology in Utrecht. These records comprised the data of patients in SPT who had an appointment for the re‐evaluation of their periodontal status. The data were entered into a specially designed Microsoft Excel spreadsheet (Version 2005). Adult periodontitis patients who had completed APT and had been following the SPT programme for at least 1 year were included in the study and patients who were younger than 36 years old were excluded.^23^, ^24^

All procedures performed in relation to the treatment of patients were in accordance with the quality standards of the Clinic for Periodontology Utrecht and with the 1964 Helsinki declaration and its later amendments.^25^ The obtained data were anonymized and, consequentially, irreversibly de‐identified. This made the revealing of any information that could be related to a specific individual impossible. In advance of the study, patients gave their permission for data related to their treatment to be anonymously used for further analysis. According to the judgment of the Ethical Committee of the Academic Centre for Dentistry Amsterdam (ACTA), this study (registered under protocol number 202010) complies with the relevant ethical guidelines.

### 2.2 Periodontal therapy

Periodontal treatment was provided according to a strict protocol described in a previous study by De Wet et al.^6^ In short, general dental practitioners refer patients to a clinic because of periodontal disease. All patients receive comparable periodontal therapy, although this is customized to individual needs. At the indication of the periodontist, this therapy can include support with systemic antibiotic therapy and/or surgical periodontal therapy. In this study, patients were entered into the SPT programme after APT when the periodontal condition had reached a level of improvement that was judged by the periodontist to be the best obtainable and which could supposedly be maintained by the patients when they entered the SPT programme. SPT started with a recall appointment with a dental hygienist every three months, which, over time, could be adapted to every four, five or six months according to the periodontal stability as judged by the periodontist. At each recall appointment, the condition of the patient's periodontium and their level of oral hygiene were checked. Dental plaque was revealed by an erythrosine solution, after which personalized oral hygiene instructions were provided. This was followed by supra‐ and sub‐gingival debridement and full‐mouth polishing. During SPT, the patients also attended re‐evaluation appointments with the periodontist at regular intervals.

### 2.3 Data collection

The following variables for this study were obtained as recorded at the time of evaluation following APT (T0) and at the latest re‐evaluation of SPT (T1):

- Number of teeth.
- Number of PPD of >5 mm.
- PESA in cm^2^.^15^
- BOP as a percentage.^26^

PPD and BOP were recorded at six sites per tooth, PESA was calculated per tooth. Besides these variables, the patients’ characteristics (i.e. gender, age at intake, smoking status, the total number of years in SPT, and the latest advised interval between SPT appointments) were collected.

### 2.4 Data management and statistical analysis

Data were transferred into SPSS (IBM SPSS Statistics for Windows, Version 25.0, Released 2017; IBM Corporation) for the statistical analyses. At first, the descriptive statistics involved mean values, standard deviations and percentages for each time point (T0 and T1). These descriptive statistics were also done for the population categorized by gender, age at intake and smoking status. This was furthermore performed for the total number of years in SPT, as it was categorized to an ordinal variable by ordering patients into 5‐year blocks (1–5, 6–10, 11–15, 16–20, 21–25, 26–30).

The Shapiro‐Wilk test and Lilliefors‐corrected Kolmogorov‐Smirnov test were conducted to test the normality of the measured clinical variables at T0 and T1. In the case of statistical significance, nonparametric tests were used to analyze the data. The mean differences between the time points were calculated for the clinical parameters, and nonparametric statistical analyses were performed using the Wilcoxon test. To reveal possible differences between the SPT duration categories, the Kruskal‐Wallis test was performed. Furthermore, differences between the categories (i.e. gender, age at intake, smoking status and the number of years in SPT) were analyzed using the Mann‐Whitney U test. Annual TL ranging from 0.11 to 0.24 teeth,^27^ a PPD of ≤5 mm,^28^, ^29^ and a PPD of ≤5 mm combined with a BOP of <10%^30^ were used as clinical endpoints for the success of the periodontal therapy. P‐values were considered to be statistically significant when *p* ≤ 0.05.

## 3 RESULTS

### 3.1 Demographics

In total, 1012 patients who were undergoing SPT visited the clinic in 2017 for a re‐evaluation of their periodontal status, and a selection of their data was entered into the database. The data of 19 patients (2%) were incomplete and were, therefore, excluded from the analysis. Subsequently, the data of 993 patients were suitable for this retrospective analysis (Table 1). The mean age of the patients at intake was 50.93 years (range 36–80). Based on the median age (50), a sub‐analysis was performed between 502 (51%) patients aged ≤50 years and 491 (49%) patients aged >50 years. The gender distribution was 45% males and 55% females, 23% of whom were considered to be smokers according to their smoking status (Table 1).

**TABLE 1:** Main description of characteristics of the population at intake (*n* = 993).

Characteristics	
Number of people (*n*)	993
Gender *n* (%)	
Male	444 (45)
Female	549 (55)
Smoking status at intake *n* (%)	
Non‐smoker	760 (77)
Smoker	233 (23)
Age at intake (years)	
Mean (SD)	50.93 (8.94)
Range	36–80
Median	50
≤ 50 years *n* (%)	502 (51)
> 50 years *n* (%)	491 (49)

%, percentage in parenthesis; SD, standard deviation in parenthesis.

As shown in Table 2, the range of patients’ number of years in SPT was 1–30 years. The number of patients categorized as having undergone 1–5 years of SPT was 500 (50%), with a mean age at intake of 52.16 years. The cohort of patients 6–10 years in SPT consisted 287 (29%), with 51.32 years as mean age at intake. Another 182 (18%) patients, with a mean age at intake of 47.66 years had received 11–15 years of SPT (Table 2). The number of patients with 16–30 years of SPT was relatively low. Therefore, these categories of SPT duration were not further analyzed. The latest advised interval of SPT was an average of 3.94 months for a total of 993 patients (Table 3).

**TABLE 2:** Main description of characteristics of patients (*n* = 993) categorized by the total number of years in SPT‐categories.

	Years in SPT (supportive periodontal therapy)					
	1–5	6–10	11–15	16–20	21–25	26–30
Number of people *n* (%)	500 (50)	287 (29)	182 (18)	13 (1)	9 (1)	2 (0)
Gender *n* (%)						
Male	245 (25)	126 (13)	65 (7)	5 (1)	2 (0)	1 (0)
Female	255 (26)	161 (16)	117 (12)	8 (1)	7 (1)	1 (0)
Smoking status at intake *n* (%)						
Non‐smoker	382 (38)	221 (22)	139 (14)	11 (1)	5 (1)	2 (0)
Smoker	118 (12)	66 (7)	43 (4)	2 (0)	4 (0)	0 (0)
Age at intake mean (SD)	52 (9)	51 (9)	48 (7)	45 (7)	46 (11)	45 (12)

**TABLE 3:** The number of years in SPT and the latest advised recall frequency (in months) for the different categories.

	Number of years in SPT	Latest advised recall frequency (in months)
	Mean (SD)	Mean (SD)
Total population (*n* = 993)	6.45 (4.41)	3.94 (2.46)
Male (*n* = 444)	6.04 (4.18)	4.05 (2.59)
Female (*n* = 549)	6.79 (4.57)	3.86 (2.35)
Non‐smoker (*n* = 760)	6.44 (4.37)	4.00 (2.45)
Smoker (*n* = 233)	6.49 (4.57)	3.77 (2.49)
Age at intake ≤50 (*n* = 502)	7.04 (4.73)	4.12 (2.68)
Age at intake >50 (*n* = 491)	5.86 (3.99)	3.77 (2.20)
SPT 1–5 years (*n* = 500)	2.87 (1.11)	4.40 (3.22)
SPT 6–10 years (*n* = 287)	7.87 (1.42)	3.48 (1.27)
SPT 11–15 years (*n* = 182)	12.23 (1.05)	3.47 (0.88)

SPT, supportive periodontal therapy; SD, standard deviation in parenthesis.

### 3.2 Success criteria

At the evaluation of APT, 36% of the patients had a PPD of ≤5 mm (absence of PPD >5 mm). If <10% BOP had been added as an endpoint for success to a PPD of ≤5 mm, only 16% of the patients would have reached this level (Table 4). During SPT (T0–T1), a mean increase of 0.77 in the number of pockets of >5 mm was found (*p* < 0.001; Table 5) and resulted in 3.61 ± 5.35 (range 0–45) pockets of >5 mm at the evaluation of SPT (T1). A mean increase of 4% (*p* < 0.001) was found during SPT, resulting in a mean BOP of 20% at the evaluation of SPT (Table 5). For the total patient population, a mean increase in the PESA of 0.26 cm^2^ (*p* < 0.001) was found during the observational period (T0‐T1), which resulted in a PESA value of 16.64 cm^2^ at T1 (Table 5).

**TABLE 4:** Success of treatment defined as PPD ≤5 mm and BOP <10% in addition to PPD ≤5 mm.

	At evaluation of APT (T0)		At evaluation of SPT (T1)	
	Success for PPD (%)	Success for PPD with BOP <10% (%)	Success for PPD (%)	Success for PPD with BOP <10% (%)
Total population (*n* = 993)	36	16	33	10
Male (*n* = 444)	32	13	29	9
Female (*n* = 549)	39	18	36	11
Non‐smoker (*n* = 760)	39	17	37	11
Smoker (*n* = 233)	27	12	20	5
Age at intake ≤50 (*n* = 502)	37	15	34	10
Age at intake >50 (*n* = 491)	35	16	32	10
SPT 1–5 years (*n* = 500)	34	17	30	9
SPT 6–10 years (*n* = 287)	41	16	37	10
SPT 11–15 years (*n* = 182)	32	12	32	10

APT, active periodontal therapy; SPT, supportive periodontal therapy; PPD, probing pocket depth, recorded at six sites per tooth; BOP,^26^ bleeding on probing, recorded at six sites per tooth.

**TABLE 5:** Mean (SD) and range of clinical measured variables at T0 (at evaluation following APT) and T1 (at evaluation of SPT) and comparison of the mean differences of clinical measured variables between T0 (at evaluation following APT) and T1 (at evaluation of SPT).

	T0 (*n* = 993)		T1 (*n* = 993)		Effect SPT (T0‐1) (*n* = 993)		*p*‐value^a^
	Mean (SD)	Range	Mean (SD)	Range	Mean (SD)	Range	
Number of teeth	25.08 (3.72)	2.0–32.0	24.24 (4.03)	2.0–32.0	−0.84 (1.60)	−14 – 0	≤ 0.001
Number of pockets >5 mm	2.84 (4.16)	0.0–34.0	3.61 (5.35)	0.0–45.0	0.77 (4.72)	−21 – 39	≤ 0.001
PESA (cm^2^)	16.38 (3.48)	3.24–30.68	16.64 (3.73)	2.94–32.35	0.26 (2.75)	−13.46 – 12.56	≤ 0.001
BOP (%)	15.97 (11.04)	0.0–87.0	20.17 (12.32)	0.0–75.0	4.20 (12.92)	−53.0 – 51.0	≤ 0.001

SPT, supportive periodontal therapy; PESA,^15^ periodontal epithelial surface area, calculated per tooth; Number of pockets, recorded at six sites per tooth; BOP,^26^ bleeding on probing, recorded at six sites per tooth; SD, standard deviation in parenthesis.

^a^ Wilcoxon test.

At the evaluation of APT (T0), the mean number of teeth was 25.08 for the total patient population (Table 5). The overall average TL during the observational period was 0.84 (*p* < 0.001; Table 5), with a calculated annual mean TL of 0.15.

### 3.3 Duration of SPT

Patients categorized as having undergone 1–5 and 6–10 years of SPT showed, on average, an increase in the number of pockets of >5 mm, BOP, and PESA during SPT. However, these results were not found for patients who had received 11–15 years of SPT. At T0, the number of pockets of >5 mm was 2.91 and 2.34, the PESA was 16.71 cm^2^ and 16.03 cm^2^, and the BOP was 14% and 17% for those with 1–5 and 6–10 years of SPT, respectively. At T1, those who had received 1–5 and 6–10 years of SPT showed 3.81 (*p* ≤ 0.001) and 3.53 (*p* ≤ 0.001) pockets of >5 mm, 17.09 cm^2^ (*p* ≤ 0.001) and 16.34 cm^2^ (*p* ≤ 0.05) PESA, and 20% (*p* ≤ 0.001) and 21% (*p* ≤ 0.001) BOP, respectively. Between the SPT duration categories, the number of pockets of >5 mm (*p* = 0.182) and the BOP percentage (*p* = 0.581) did not differ at the re‐evaluation of the periodontal status during SPT. TL showed an increase (*p* < 0.001) for each of the SPT duration categories during the observational period. When patients who had received 1–5 years of SPT (TL = 0.54) were compared with those with 6–10 years (TL = 0.92) and those with 11–15 years of SPT (TL = 1.31), TL differed significantly (*p* < 0.001). The lowest annual TL was found for those who had spent the longest time in SPT (0.11).

### 3.4 Patient characteristics

At the evaluation of APT, 32% of the 444 males and 39% of the 549 females had a PPD of ≤5 mm. This was 13% and 18%, respectively, when a PPD of ≤5 mm was combined with a BOP of <10% (Table 4). Overall, the number of pockets of >5 mm differed between the males (1.27) and females (0.36; *p* = 0.015). Of the 233 smokers and 760 non‐smokers, at T0, a PPD of ≤5 mm was found for 27% and 39% of patients, respectively. When in addition to a PPD of ≤5 mm a BOP of <10% was selected, 12% and 17% of the smokers and non‐smokers, respectively, had this condition at the evaluation of APT (Table 4). The TL (*p* = 0.002) and increase in the number of pockets of >5 mm (*p* = 0.005) were found to differ between the smokers and non‐smokers. At the evaluation of APT, of the 502 patients aged ≤50 and the 491 patients aged >50 years at intake, 37% and 35% had pockets of ≤5 mm, respectively. Furthermore, at the evaluation of APT when the success of treatment defined as PPD of ≤5 mm combined with a BOP of <10%, it was found that 15% of the patients at intake aged ≤50 and 16% of the patients aged >50 reached this (Table 4). During SPT, TL and the increase in BOP did not differ between the categories.

## 4 DISCUSSION

### 4.1 Summary of the findings

This retrospective analysis considered the stability and success of SPT in chronic periodontitis patients aged ≥36 years, comparing the outcomes at the evaluation of APT to those at the latest SPT re‐evaluation. The results of this analysis represent a practice‐based situation in a clinic that specializes in periodontal therapy. When defining post‐treatment success as a PPD of ≤5 mm,^28^, ^29^ approximately one‐third of the total patient population reached this endpoint at the evaluation of APT. Moreover, when a BOP of <10% was used as a criterion for the success of APT in addition to a PPD of ≤5 mm, a lower percentage of the population was found to have reached this goal.^30^ During the SPT treatment, an overall increase in TL, the number of pockets of >5 mm, PESA and BOP was found for the total population, although at the re‐evaluation of SPT, the achieved APT success was ‘fairly’ well‐maintained. The present analysis did not find an association between age at intake and the success of therapy, as the achieved success was maintained in both age categories. Smoking and male gender appeared to have a negative impact on the success of SPT.

### 4.2 The success of APT

In this analysis, successful treatment was defined as a PPD of ≤5 mm after the evaluation of APT, as reported by Badersten et al.^29^ and Van der Weijden et al.^28^ Several authors have used other endpoints, such as a PPD of 4–5 mm,^6^ <9 sites with a PPD of 5 mm,^31^ or a PPD of <6 mm^31^ to define the success of therapy. This inconsistent use of boundary values by researchers makes a comparison between studies virtually impossible. For the analyzed population, 36% of the patients were considered to have been ‘successfully treated’ when the endpoint PPD of ≤5 mm was used. The therapy could, therefore, be labelled as ‘not as effective as expected’. New defined endpoints may be more suitable for daily clinical practice and research when predicting the success of treatment, for which suggestions have been made by Feres et al.^32^ One should also keep in mind that the remission or control of periodontal disease is not necessarily reflected by PPD. PPD as a criterion for the endpoint of treatment provides limited information. Therefore, more research about other endpoints must be conducted to measure the stability of periodontitis, considering patients’ health as a parameter.^14^

According to the consensus statement of the 2017 World Workshop on the Classification of Periodontal Disease,^30^ the threshold for a clinical case of health in a successfully treated periodontitis patient should be set at ≤4 mm with no BOP. However, this continuous absence of BOP may not be attained by treated periodontitis patients.^30^ Therefore, periodontal stability is characterized by minimal (<10% of sites) BOP.^30^ If this BOP threshold had been applied in the present analysis, only 16% of the patients would have been classified as successfully treated. If this information is regarded in the context of the stability observed during SPT in this retrospective analysis, attempting to reach stricter endpoints than a PPD of ≤5 mm more teeth would have needed to be extracted. Considering that the periodontist entered the patient into SPT at a stage where the best obtainable periodontal status was reached this could have resulted in over‐treatment.^33^

A PESA score of up to 11 cm^2^ is considered to indicate healthy periodontium, according to Nesse et al.^15^ In the present analysis, scores ranging from 11 cm^2^ to 39 cm^2^ were observed in the periodontitis patients, both at T0 and T1. Consequently, the periodontal status of the total analyzed population could not be labelled as ‘healthy’. The role of the PESA measurements as endpoints of therapy or as predictors of periodontal stability does not appear to provide relevant information in this study population that can be used in daily clinical practice.

### 4.3 The success of SPT

Concerning periodontal stability, there is within both scientists and clinical professionals discussion about valid criteria. The present analysis reveals a small increase in all clinical parameters during SPT. However, as shown in Table 4, when translating these according to the predefined success criterion of a PPD of ≤5 mm, almost no difference is found between T1 (33%) and T0 (36%). Additionally, one should note that the measured success rate is relative and could be considered even higher if the analyzed population included a control group of periodontitis patients who did not enter the SPT programme. Such a comparison would, however, not be considered ethical in the case of long‐term follow‐up evaluations. Altogether, with SPT, the treatment effect as established after APT can be maintained. Patient compliance to SPT has been shown to positively influences the treatment outcomes.^34^

### 4.4 Tooth loss

There are various reasons for extractions, including the diagnosis of a tooth as ‘hopeless’ by the dental care professional. Since there is no standard definition of a ‘hopeless’ tooth, this parameter has several limitations.^32^ Teeth recorded as extracted could have been lost because of periodontal reasons but also to reasons such as trauma, endodontic failure or impossibility to restore. Nonetheless, TL is considered as a meaningful clinically measured direct outcome of therapy and is not susceptible to bias, unlike PPD and BOP.^35^ Therefore, TL can be considered a true endpoint for the success of periodontal therapy. The pattern of TL appears to be predictable according to the position in the arch.^36^ According to a recent analysis, a range of 0.11–0.24 TL per year during SPT is perceived to be an achievable endpoint value.^27^ Therefore, the present analysis, with an annual mean TL of 0.15, can be considered to have an acceptable level of TL that is also consistent with other studies.^37^ This corresponds with what is regarded as long‐term periodontal stability if patients are persistent and SPT is provided at regular intervals. Other authors^38^ have found an annual TL of 0.15 and 0.09 for 5 years and 12–14 years of follow‐up, respectively, and considered this to be ‘low incidence’. A wide range of 0.05–0.17 annual TL was found among different studies for patients in SPT with a duration of 6 to 10 years.^27^ The present analysis showed comparable differences in annual TL for the SPT duration categories. Evidently, the highest total tooth loss score was attributed to patients who had spent the longest time in SPT. This could be because more complex patients may be more committed to receiving long‐term therapy. At the same time, the lowest annual TL of 0.11 was found for patients who had spent the longest time in SPT, which corresponds with the observation of Trombelli et al.^38^ While other studies^19^, ^39^ found a higher risk of TL for periodontitis patients who were older than 55 years, of male gender, and smokers, these findings are not consistent with the results of the present analysis, which found a higher TL for smokers but did not find any age or gender differences for TL during SPT. This is similar to the results of De Wet et al.,^6^ which included a selected patient population from the same periodontal clinic that was fully adherent to the advised recall visits for a period of 9 years. All in all, the observed annual TL in the present analysis corresponds with the previously described ranges during SPT.^27^ It is, therefore, safe to state that the annual TL found can be considered to be acceptable, which is an indication of periodontal stability. However, the implications of TL are wide and the impact on the patient's quality of life is immeasurable.^2^, ^3^, ^6^, ^27^

### 4.5 SPT duration

The utilized database contained retrospective data as obtained from two points in time. The time between these points was dependent on the time over which patients had received SPT and was categorized into blocks of 5 years. As this analysis represented a practice‐based population, it included a variety of participants who may not all have been equally persistent in strictly adhering to the advised regular intervals. The SPT success rate could be related to the patients’ adherence to long‐term SPT. Adherence to SPT generally reduces over the years, and the biggest drop‐out of patients tends to occur within the first 5 years of SPT.^40^ Patients who are adherent to long‐term therapy are often more committed and have a better mindset to remain healthy.^41^, ^42^ However, like previous studies,^30^, ^31^, ^40^, ^41^, ^43^ this analysis showed that the longer the SPT duration, the higher the recall frequency (Table 3). Since the recall frequency depends on individual needs and the stability of the patients’ periodontium, the patients who adhered to long‐term SPT may have maintained a worse periodontal condition compared to the patients who dropped out. Nonetheless, as in the study of Wasserman and Hirschfeld,^44^ good long‐term response was observed, demonstrating that the level of the initial clinical parameter was not associated with the long‐term result. In the present analysis, the number of pockets of >5 mm, PESA and BOP significantly increased for patients with 1 to 5 and 6 to 10 years of SPT during the observational period. This was not the case for the patients with 11–15 years of SPT. More importantly, the percentages from this category of treatment success remained the most stable during SPT. Although gender and smoking status are important parameters that affect the outcome of SPT, they do not explain this finding, as the distribution of male gender and smokers was equal between the SPT duration categories. In short, one may conclude that SPT is successful in the long term.

### 4.6 The relationship between gender and the success of SPT

In the present analysis, males had a greater increase in the number of pockets of >5 mm compared to females during SPT. However, when the SPT duration categories were analyzed by gender, a significant gender difference was found only for patients who had undergone 1 to 5 years of SPT. The dissimilarity in SPT success between males and females may be related to a difference in adherence.^41^, ^45^ It could be that after the first 5 years of SPT, the more adherent males remained.^40^ When comparing males and females in the different SPT duration categories, one's impression is that the percentage of males who dropped out of SPT may be higher than that of females (Table 2), which is similar to the finding of a 14‐year retrospective study conducted by Demetriou et al.^46^ However, more research is necessary to obtain conclusive findings on the effect of patients’ gender and adherence with regard to the long‐term success of SPT. The drivers of this inner motivation for the patients’ persistence should be investigated not only from the periodontal aspect but also from a psychologic point of view, with input from daily clinical practice.

### 4.7 The relationship between smoking status and the success of SPT

The prevalence, severity and progression of periodontitis are affected negatively by smoking status.^18^ With regard to TL and the number of pockets of >5 mm during SPT, the findings of the present analysis showed a significant difference between smokers and non‐smokers. Earlier work that has emerged from the same periodontal clinic has already shown an association between tobacco smoking and a higher number of pockets of >5 mm.^47^ More importantly, during SPT, the achieved success in non‐smokers was better maintained than in smokers. The negative effect of smoking on the success of SPT observed in this analysis should be taken into consideration by interpreting the data.

### 4.8 Limitations

Data used for the analysis were retrospectively collected at the Clinic for Periodontology Utrecht, which is organized according to the ISO 9001:2015 standard.^48^ Because the therapy and measurements were performed within a routine clinical setting, the risk of selective reporting of results is considered to be low, although there is a possibility that the information that may have influenced the patients’ responses to SPT is incomplete. Without insight into the patients’ medical files describing the presence of systemic diseases or the awareness of unfavourable lifestyles, the analysis might have missed potential risk factors. Moreover, due to the acquisition of data during routine periodontal therapy, the periodontist was not blinded to previous patient records, which may have affected the outcome.

Although medical details such as systemic diseases may affect the severity of periodontitis and the outcome of periodontal therapy^14^, details on medical status were not entered into the data base used for this included in this study. The new classification of periodontitis^2^ highlights two risk factors of which ‘smoking’ is assessed in this paper. The other risk factor ‘diabetes’ was addressed in an earlier retrospective analysis^49^ and found to have a low prevalence among the patient population of the practice and not to be related to extent and severity of periodontitis of the patient sample.

Because the data for this analysis were gathered in 2017, the recent ‘new classification for periodontal and peri‐implant diseases and conditions^50^ was not applied. At intake, patients selected for this analysis were classified as having ‘adult periodontitis’, because, at that time, classification was performed according to Van der Velden's definitions.^23^, ^24^ Retrospective reclassification of the present patient population was not achievable.

Another limitation of this analysis could be its generalizability since the findings were obtained from a database from a clinic that is specialized in periodontal therapy. Numerous studies have demonstrated the beneficial effect of periodontal treatment and maintenance in this specific private periodontal clinic.^6^, ^28^ It is known from research that patients in private periodontal clinics compared to those treated in an academic setting show significant better stability of the periodontal condition based on less progression of periodontitis and tooth loss.^51^ The analyzed patient population may, for instance, belong to a higher socio‐economic group for whom SPT is more affordable. The results of the present analysis should, therefore, be interpreted with caution, as the findings possibly involve bias toward the potential success of SPT.

In this analysis, former smokers and non‐smokers were aggregated into the same group, yet the effect on the periodontal status of former smokers should not be ignored.^52^ If former smokers and non‐smokers had been classified into different categories, the findings of the detrimental effect of smoking behaviour on the success of SPT could have been more precise. Another factor that was not reported was whether non‐smokers started smoking—or smokers quit smoking—during SPT, yet the cessation of smoking has a positive effect on probing pocket depths.^53^, ^54^ Moreover, no data were obtained on the number of cigarettes smoked daily by smokers or the duration of their smoking habit.

### 4.9 Future research

In further research on the success of long‐term SPT, definitions of new (clinical) endpoints of APT, such as those suggested by Feres and co‐workers^32^ and also by Loos and Needleman (2020),^14^ should be assessed, as these could prove to be beneficial in describing the success of therapy and the periodontal stability during SPT. Also, the impact of patients’ medical status on the success of SPT should be addressed.

## 5 CONCLUSION

This retrospective analysis indicates that after APT, the periodontal clinical status remained ‘fairly’ stable during SPT in treated periodontitis patients aged ≥36 years. Although all clinical parameters showed a small but significant increase during SPT, the annual TL was considered to be acceptable and the obtained APT treatment success was maintained. Furthermore, this analysis did not find an association between patients’ age at intake and periodontal stability during SPT. Smoking has a negative effect both on the outcome of APT and periodontal stability during SPT.

## 6 CLINICAL RELEVANCE

### 6.1 Scientific rationale for the study

Clinical research on ensuring long‐term periodontal stability in periodontitis maintenance patients is limited. Therefore, an evaluation of the stability and success of SPT in chronic periodontitis patients in a clinic restricted for periodontology is of scientific interest.

### 6.2 Principle findings

A small increase was found for all clinical parameters and an annual TL of 0.15. During SPT, on average, the treatment effect obtained after APT was successfully maintained.

### 6.3 Practical implications

This analysis demonstrates that regular SPT is successful in maintaining a stable periodontal condition in adult periodontitis patients.

## CONFLICT OF INTEREST

Van der Weijden is the owner of the Clinic for Periodontology, Utrecht, the Netherlands. All other authors state that they have no further conflict of interest.

## AUTHOR CONTRIBUTION

All authors gave their final approval and agreed to be held accountable for all aspects of the work, ensuring integrity and accuracy.

Schoenmakers: contributed to analysis and interpretation and drafted the manuscript.

Willems: contributed to analysis and interpretation and drafted the manuscript.

Slot: contributed to analysis and interpretation and critically revised the manuscript.

Van der Weijden: contributed to conception and design, analysis and interpretation, and critically revised the manuscript.

## Data Availability

The data that support the findings of this study are available from the corresponding author upon reasonable request.
